# The Mitochondrial A10398G Polymorphism, Interaction with Alcohol Consumption, and Breast Cancer Risk

**DOI:** 10.1371/journal.pone.0005356

**Published:** 2009-04-24

**Authors:** Annamaria Pezzotti, Peter Kraft, Susan E. Hankinson, David J. Hunter, Julie Buring, David G. Cox

**Affiliations:** 1 Program in Molecular and Genetic Epidemiology, Epidemiology Department, Harvard School of Public Health, Boston, Massachusetts, United States of America; 2 Biostatistics Department, Harvard School of Public Health, Boston, Massachusetts, United States of America; 3 Channing Laboratory, Department of Medicine, Brigham and Women's Hospital and Harvard Medical School, Boston, Massachusetts, United States of America; 4 Division of Preventive Medicine, Brigham and Women's Hospital and Harvard Medical School, Boston, Massachusetts, United States of America; Innsbruck Medical University, Austria

## Abstract

Polymorphisms in the mitochondrial genome are hypothesized to be associated with risk of various diseases, including cancer. However, there has been conflicting evidence for associations between a common polymorphism in the mitochondrial genome (A10398G, G10398A in some prior reports) and breast cancer risk. Reactive oxygen species, a by-product of mitochondrial energy production, can lead to oxidative stress and DNA damage in both the mitochondria and their cells. Alcohol consumption, which may also lead to oxidative stress, is associated with breast cancer risk. Therefore, we hypothesized that polymorphisms in the mitochondrial genome interact with alcohol consumption to alter breast cancer risk. We genotyped the A10398G polymorphism in a case-control study nested within the Nurses' Health Study (NHS, 1,561 cases, 2,209 controls). We observed an interaction between alcohol consumption (yes/no) and A10398G on breast cancer risk (p-int = 0.03). The risk associated with alcohol consumption was limited to carriers of the 10398G allele (Odds Ratio 1.52, 95% Confidence Interval 1.10–2.08 comparing drinkers to non-drinkers). However, we were unable to replicate these findings in the Women's Health Study (WHS, 678 cases, 669 controls), although the power to detect this interaction in the WHS was low (power = 0.57). Further examination of this interaction, such as sufficiently powered epidemiological studies of cancer risk or associations with biomarkers of oxidative stress, may provide further evidence for GxE interactions between the A10398G mitochondrial polymorphism and alcohol consumption on breast cancer risk.

## Introduction

Mitochondria are very important cellular organelles, playing active roles in various functions, such as energy balance and cell cycle control. In addition, by transforming energy into a form that the cell can use, mitochondria produce reactive oxygen species (ROS) that can lead to damage of key components of the cell, particularly proteins and DNA. All of these facets of mitochondrial biology make them of interest with respect to cancer biology.

As mitochondria were once symbiotic bacteria living within early eukaryotes, they still carry their own genome, separate from the nuclear genome. While many genes have been shared between the mitochondrial and nuclear genomes of humans over evolutionary history, the mitochondrial genome still retains many of the genes required for its proper functioning. Like the nuclear genome, the mitochondrial genome carries polymorphic sequences. Interestingly, it is hypothesized that polymorphisms in the mitochondria that cause the mitochondria to be less efficient in energy exchange, therefore creating more heat and reactive oxygen species, allowed early humans an adaptive advantage when migrating to cooler climates, such as from Africa to Europe [1], and advances in genotyping and sequencing technology have led to greater understanding and fine-scale resolution of human migrations based on mitochondrial genetics [2]. Other polymorphisms have more recently been investigated for association with various diseases, including diabetes [1], [3]–[6], Parkinson's disease [7]–[9], Alzheimer's disease [8], [10]–[12], and breast cancer [13]–[16].

The nonsynonymous A10398G (T114A) polymorphism in the ND3 (NADH dehydrogenase subunit) gene of the mitochondrial genome is one of the most extensively studied mitochondrial polymorphisms. There is no prior knowledge of this polymorphism affecting mitochondrial function, however it has been associated with neurodegenerative diseases such as Parkinson's[7] disease , Friedrich's ataxia [17], and ALS[18]. Evidence of an association between this polymorphism and breast cancer risk has been inconclusive [13]–[16]. Therefore, we studied this polymorphism in the case-control study of breast cancer nested in the prospective Nurses' Health Study (NHS) cohort, consisting of 1,561 cases and 2,209 controls. We also hypothesized that alcohol consumption, which increases oxidative stress and is associated with breast cancer risk[19], would modify the association between A10398G and breast cancer risk. Upon observing an interaction between alcohol consumption and A10398G on breast cancer risk, we sought to confirm this finding in the Women's Health Study (WHS), where 678 cases and 669 controls were successfully genotyped.

## Results

Baseline characteristics of both the NHS and WHS are reported in Table 1. We did not observe any association between the A10398G polymorphism in the mitochondrial ND3 gene and breast cancer risk in either the NHS (Odds Ratio (OR) 1.01, 95% Confidence Interval (CI) 0.85–1.19) or WHS (OR 0.94, 95% CI 0.72–1.22) (Table 1.). Results were similar between conditional and unconditional analyses, so we will present unconditional analyses in order to include all samples with available data. There was no evidence of confounding by any of the covariates included in the models, however we have chosen to include them in all analyses to control for even minor confounding.

**Table 1:** Baseline Characteristics of Study Participants from the Nurses' Health Study and Women's Health Study

	Nurses' Health Study		Women's Health Study	
	Cases (N = 1561)	Controls (N = 2209)	Cases (N = 678)	Controls (N = 669)
Age (St.Dev.)	56.9 (6.96)	57.8 (6.87)	60.5 (7.60)	60.3 (7.58)
A10398G G carriers (%)	319 (20.4)	447 (20.2)	149 (22.0)	151 (22.6)
Mean alcohol consumption g/day (St. Dev.)	5.28 (9.13)	5.10 (8.82)	4.70 (8.79)	4.39 (8.90)
Alcohol consumption ever/never (%)	983 (63.0)	1358 (61.5)	390 (57.5)	362 (54.1)

Major ancestry was asked in the Nurses' Health Study by questionnaire in 1992 and 98% of the participants who responded to this question are self described Caucasians. Furthermore, 659 (18% of the total) of the subjects described themselves as South European/Mediterranean, 260 (7% of the total) as Scandinavian, and 2603 as Other Caucasian (73% of the total). In these sub-categories, the G allele of A10398G is present at 18.5, 18.1, and 19.7% respectively. 28 subjects described themselves as African-American, 11 as Hispanic and 14 as Asian. In these sub-categories, the G allele of A10398G is present at 89.3, 9.1, and 64.3% respectively.

We did observe, however, an interaction between A10398G and alcohol consumption (alcohol coded as non-drinkers/drinkers, p-interaction = 0.03) in the NHS. P-values from both the Wald and Likelihood Ratio Tests (LRT) were similar (identical to the third decimal place at 0.026), as expected. In cross-tabulated analyses, women carrying the variant G allele and not consuming alcohol were at decreased risk of breast cancer as compared to women not carrying the variant and not consuming alcohol (OR 0.79, 95% CI 0.60–1.04, Table 2). The association between alcohol consumption and breast cancer risk among carriers of the G allele of A10398G in the NHS was 1.52 (95% CI 1.10–2.08, Table 2). Restricting these analyses to self-described Caucasian participants did not significantly alter the results, OR 1.56, 95% CI 1.12–2.16.

**Table 2:** Interaction between A10398G, Alcohol Consumption, and Breast Cancer Risk in the Nurses' Health Study (NHS) and the Women's Health Study (WHS)

**NHS**		**Cases (%)**	**Controls (%)**	**OR** * **(95% CI)**
	A10398, non-Drinkers	475 (30.5)	662 (30.0)	1.00 (Ref.)
	A10398, Drinkers	767 (49.2)	1100 (49.8)	1.02 (0.87–1.19)
	G10398, non-Drinkers	103 (6.6)	189 (8.6)	0.79 (0.60–1.04)
	G10398, Drinkers	216 (13.9)	258 (11.7)	1.18 (0.95–1.48)
A10398 Carriers	Non-Drinkers	475 (38.2)	662 (37.6)	1.00 (Ref.)
	Drinkers	767 (61.8)	1100 (62.4)	0.96 (0.83–1.12)
G10398 Carriers	Non-Drinkers	103 (32.3)	189 (42.3)	1.00 (Ref.)
	Drinkers	216 (67.9)	258 (57.7)	1.52 (1.10–2.08)
**WHS**		**Cases (%)**	**Controls (%)**	**OR** ** **(95% CI)**
	A10398, non-Drinkers	225 (33.2)	245 (36.6)	1.00 (Ref.)
	A10398, Drinkers	304 (44.8)	273 (40.8)	1.22 (0.95–1.57)
	G10398, non-Drinkers	63 (9.3)	62 (9.3)	1.08 (0.73–1.62)
	G10398, Drinkers	86 (12.7)	89 (13.3)	1.03 (0.72–1.46)
A10398 Carriers	Non-Drinkers	225 (42.5)	245 (47.3)	1.00 (Ref.)
	Drinkers	304 (57.5)	273 (52.7)	1.22 (0.95–1.57)
G10398 Carriers	Non-Drinkers	63 (42.3)	62 (41.1)	1.00 (Ref.)
	Drinkers	86 (57.7)	89 (58.9)	0.96 (0.60–1.53)

* Unconditional logistic regression controlling for fasting status, date and time of blood draw, age, body mass index (at blood draw and age 18), menopausal status, family history of breast cancer and history of benign breast disease. P-interaction = 0.03

** Unconditional logistic regression controlling for age, history of benign breast disease and family history of breast cancer. P-interaction = 0.95

We did not observe this interaction in the WHS (Table 2). However, the prevalence of alcohol consumption in the WHS (Table 1) was lower than in the NHS, and the power to detect this interaction in the WHS is low (power = 0.57, alpha = 0.05). Combining data from both the NHS and WHS by meta-analyses reduced the point estimate of risk of breast cancer associated with alcohol consumption among carriers of the G allele of A10398G to 1.33, 95% CI 1.04–1.71.

## Discussion

The mitochondria's role in various cellular processes, including production of energy and ROS, DNA damage and repair, and cell cycle control make them likely candidates to play a role in risk of cancer. In addition, the mitochondrial genome contains polymorphisms and is transmitted only from the mother to offspring. Therefore, mitochondrial polymorphisms are strong candidates as markers of risk for many diseases, including breast cancer.

In our discussion, we will use the terms “Caucasian” or “Hispanic” if this is how either the study subjects (with respect to the NHS) describe themselves, or how the participants were described in prior publications. It should be noted that the A10398G polymorphism is of great importance with respect to mitochondrial phylogeny, being relatively less frequent in West Eurasian populations as opposed to African or Asian lineages[2] and therefore population stratification with respect to this variant should be discussed. A substantial subset of the breast cancer cases and controls (∼1150 cases and one of their matched controls) from the Nurses' Health Study has participated in the first stage of a genome wide scan. In principal component analyses of over 500,000 SNPs from this scan, no population substructure (other than that which corresponds to self-described ancestry into “Caucasian”, “Hispanic”, “Asian”, and “African-American”) was observed[20]. Similarly, there is no heterogeneity in allele distributions between the self-reported sub-populations reported by the cases and controls. Further restricting our analyses to self-described Caucasians did not substantially alter our results (OR 1.56, 95% CI 1.12–2.17 for alcohol consumption among carriers of the G allele of A10398G). In order for population stratification to explain the interaction observed in the NHS, such substructure would need to be associated with both breast cancer and alcohol consumption. Heterogeneity, either in exposure to alcohol, genetic background (and population substructure), and baseline breast cancer risk could explain the lack of replication of the interaction observed in the NHS by the WHS. However, the most likely explanation for the lack of replication is random variation in these variables, and either a false positive interaction in the NHS or a false negative interaction in the WHS.

One limitation of this study is that having genotyped only one mitochondrial variant, we are unable to categorize the participants of this study into well defined mitochondrial haplogroups as done by Bai et al.[16] However given the prior work published on the non-synonymous A10398G (T114A) polymorphism in the mitochondrial NADH dehydrogenase subunit 3 (ND3) and breast cancer risk [13]–[16] coupled with the possibility of its putative function, we feel that the *a priori* hypothesis of an interaction between alcohol consumption and the A10398G polymorphism on breast cancer risk is relatively strong.

The A allele of the A10398G polymorphism has been associated with an increase in risk of breast cancer in African-Americans (654 cases, 605 controls, OR 1.60 (95% CI 1.10–2.93), but not among Caucasians (879 cases, 760 controls, OR 1.03 (95% CI 0.81–1.31) [14]. In a smaller case-control study (124 cases and 273 controls) of women from the Indian sub-continent, Darvishi et al. also observed an increase in breast cancer risk with the A allele (OR 1.73 (95% CI 1.13–2.66) [15]. While Darvishi et al. do not report specific allele distributions in their population, the prevailing haplogroups in their Indian population are more closely related to the African population with respect to the allele distribution of the A10398G polymorphism. Bai et al. studied 29 mtDNA variations in a small case control study of breast cancer (156 women with familial breast cancer and 260 controls). They observed an inverse association between haplogroup U (which contains the 10398A allele) and breast cancer risk [16]. Recently, Setiawan et al. did not observe an association between this polymorphism and breast cancer risk among African-American populations in California (1456 cases and 978 controls) [13].

The Nurses' Health Study has greater than 80% power to detect a 26% increase (OR = 1.26) in risk between the A10398G polymorphism and breast cancer at an alpha of 0.05. Therefore, we can exclude any association between this polymorphism and breast cancer risk with an odds ratio above 1.26, such as those first observed in African-Americans (OR = 1.60) and Indians (OR = 1.73) in our study population. We have limited power to detect, however, more moderate (OR<1.26) associations between this polymorphism and breast cancer risk.

Our findings of an interaction between alcohol consumption and A10398G in the Nurses' Health Study are of interest. Alcohol is a well established breast cancer risk factor [19]. A metabolite of alcohol, acetyladehyde, is a reactive oxygen species, and increases oxidative stress [21]–[23]. Given the prior epidemiologic data of associations between A10398G and various diseases, it is hypothesized that the G allele has lower potential to generate reactive oxygen species. This is in concert with our observation that women in the Nurses' Health Study who do not consume alcohol and carry this allele have a 21% lower risk of breast cancer as compared to non-drinkers who do not carry the allele. An alternative way of examining these findings with more relevance to public health is to look at the association between alcohol consumption within genotype categories. Among women carrying the G allele, alcohol consumption is associated with a 56% increase in breast cancer risk in the Nurses' Health Study.

Further examination of the interaction between alcohol consumption and the A10398G polymorphism of the mitochondrial genome on breast cancer risk could refine our observations. While it is hypothesized that A10398G alters the production of reactive oxygen species in the mitochondria, there is no *in vitro* evidence to date to support this hypothesis. Therefore the interaction that we have observed between this polymorphism, which may well be nothing more than a marker of other mitochondrial variation and have no functional relevance specifically, and alcohol consumption on breast cancer risk could be due to other mitochondrial polymorphisms which occur in common with A10398G. Epidemiologic studies are generally ill-suited to describe a causal marker when multiple correlated markers, all with little if any known functional relevance to the phenotype in question, exist.

Additional epidemiologic evidence such as evidence for interactions between this polymorphism and/or other variants with which it is correlated and alcohol consumption in other breast cancer studies would also be of interest. However, the studies would need to have sufficient power to detect such an interaction. Similarly, as alcohol consumption is associated with other diseases (presumably through increased oxidative stress), including other cancer sites, it is possible that similar interactions could be observed with risk of these diseases and should be explored. Alternatively, interactions between this polymorphism and alcohol consumption on markers of oxidative stress would also provide biological evidence supporting our observations.

In conclusion, we have detected an interaction between the A10398G polymorphism in the human mitochondrial genome and alcohol consumption associated with breast cancer risk. In the Nurses' Health Study, drinkers who carry the 10398G allele are at a 56% higher risk of breast cancer than non-drinkers with the same allele. This interaction was not observed in the Women's Health Study; however there was insufficient power to detect this association in this study. Upon combining results from the NHS and WHS, the risk of breast cancer associated with alcohol consumption among carriers of the G allele of A10398G was reduced to 33% (OR 1.33, 95% CI 1.04–1.71) Further examination of the possible interaction between alcohol consumption and the A10398G polymorphism, or other variants for which it is a marker, on breast cancer risk is needed to further define this association. Additionally, understanding of the biological significance, if any, related to the A10398G polymorphism (or other variants co-existing in the mitochondrial genome with A10398G) and how it/they could interact with alcohol consumption would also be of great interest.

## Materials and Methods

The Nurses' Health Study nested breast cancer case control study (cases, n = 1,561; controls, n = 2,209) is derived from 32,826 women who were free of diagnosed breast cancer at blood collection in 1989 and 1990, and were followed for incident disease until May 31, 2004. Medical records were used to confirm the diagnoses in women who reported a diagnosis of breast cancer on the biennial questionnaires. Control subjects were matched to cases based on age, menopausal status, recent hormone replacement therapy, and blood-draw specific variables (such as date and time of day). Questionnaires were used to determine the subject's major ethnic group (Caucasian, Hispanic, African-American, Asian), as well as further detail within Europe (Mediterranean/Southern European, Scandinavian, Other Caucasian). The nested breast cancer case control study in the Harvard Women's Health Study began in 1993 when 28,263 women provided blood samples and were followed for incident disease until March 7, 2000, with 702 cases and 703 controls selected using the same criteria as in the NHS. Detailed descriptions of these cohorts have been published previously, and a brief summary of the subjects in these analyses are in Table 1.

The A10398G polymorphism in the mitochondrial genome was genotyped using Taqman technology (Applied Biosystems, Foster City, CA). Primer and probe sequences as well as cycling conditions are available from the authors. Internal blinded quality control (QC) samples were 100% concordant, and greater than 95% of samples yielded genotypes. Hardy-Weinberg equilibrium tests are not valid for mitochondrial SNPs, and therefore were not assessed, and no heteroplasmy (detected as heterozygous samples) was observed.

We included possible confounding factors, including body mass index (BMI), family history of breast cancer, self reported history of benign breast disease, age, and menopausal status in all models in the Nurses' Health Study. We also used unconditional logistic regression with the Women's Health Study, controlling for age, family history of breast cancer, and self reported history of benign breast disease. Tests for interactions were performed using Wald tests that include main effects and interaction terms in the model, and likelihood ratio testing, comparing the fit of the model with only main effects to the fit of the model with both main effects and interaction terms. All statistical analyses were carried out using SAS 9.1 (SAS Institute, Cary NC), with the exception of power calculations which were performed in the Quanto software[24] and pooled analyses which were performed using the rmeta package in R. We analyzed the association between A10398G and alcohol intake and breast cancer risk using unconditional logistic regressions using Proc Logistic to include the greatest number of case-control sets in interaction analyses. Alcohol consumption was classified as ever/never due to the low level of consumption in both cohorts. Cross-tabulated analyses were carried out by creating indicator variables for each cell of the cross-classified table, and fitting these indicator variables in the model. We additionally examined the effect of alcohol consumption in strata according to allele carrier status.

### Ethics statement

Informed written consent was obtained from all participants, and this study was approved by the Institutional Review Board of the Brigham and Women's Hospital.
